# Supplementary material for: Adult Feeding Experience Determines the Fecundity and Preference of the Henosepilachna vigintioctopunctata (F.) (Coleoptera: Coccinellidae)
Source: Biology (Basel). 2024 Apr 9;13(4):250. doi: 10.3390/biology13040250 (PMC11048397; doi:10.3390/biology13040250)
Supplement: Supplementary file 1 [file biology-13-00250-s001.zip › biology-2927708-supplementary.pdf]

Table S1 The numbers of female and male replicates across different treatments

	Treatment								
	PP	PT	PE	TP	TT	TE	EP	ET	EE
Female	35	33	30	40	42	44	34	38	34
Male	32	33	36	45	41	41	39	30	38

The preadult survival rate of *H. vigintioctopunctata* on eggplant, tomato, and potato will dictate the number of replicates required in the match-mismatch experiments. Simultaneously, during the experiment, cases of adult escapes or accidental mortality must be documented, and the data for these adults removed.
